# 

**Published:** 1907-02-15

**Authors:** 


CONTENTS

| | | |
|---|---|---|
| **Event and Comment** | | 55 |
| Gold Inlays | *By C. H. Worboys, D.D.S.* | 59 |
| Modern Dental Remedies | *By H. T. Loeffler, B.S., D.D.S.* | 68 |
| With Our Editors | | 76 |
| Bibliography | | 89 |
| Indents | | 91 |
| Announcements | | 104 |

